# Dimethylmyricacene: An In Vitro and In Silico Study of a Semisynthetic Non-Camptothecin Derivative Compound, Targeting Human DNA Topoisomerase 1B

**DOI:** 10.3390/cells11213486

**Published:** 2022-11-03

**Authors:** Alessio Ottaviani, Federico Iacovelli, Joshua Welsch, Blasco Morozzo della Rocca, Alessandro Desideri, Mattia Falconi, Laurent Calcul, Bill J. Baker, Paola Fiorani

**Affiliations:** 1 Department of Biology, University of Rome Tor Vergata, 00133 Rome, Italy; 2 Department of Onco-Hematology, Gene and Cell Therapy, Bambino Gesù Children’s Hospital-IRCCS, 00146 Rome, Italy; 3 Department of Chemistry, University of South Florida, Tampa, FL 33620, USA; 4 Institute of Translational Pharmacology, National Research Council, CNR, 00133 Rome, Italy

**Keywords:** human topoisomerase 1B, dimethylmyricacene, natural compound inhibition

## Abstract

Human topoisomerase 1B regulates the topological state of supercoiled DNA enabling all fundamental cell processes. This enzyme, which is the unique molecular target of the natural anticancer compound camptothecin, acts by nicking one DNA strand and forming a transient protein–DNA covalent complex. The interaction of human topoisomerase 1B and dimethylmyricacene, a compound prepared semisynthetically from myricanol extracted from *Myrica cerifera* root bark, was investigated using enzymatic activity assays and molecular docking procedures. Dimethylmyricacene was shown to inhibit both the cleavage and the religation steps of the enzymatic reaction, and cell viability of A-253, FaDu, MCF-7, HeLa and HCT-116 tumor cell lines.

## 1. Introduction

Human DNA topoisomerase 1B (hTop1) is a ubiquitous enzyme, essential in mammals [1]. HTop1 solves all topological problems that take place during processes, such as DNA replication, transcription and chromosome segregation, in order to allow cell proliferation [2]. This monomeric enzyme of 765 amino acids with a molecular weight of 91 kDa is divided into four domains. HTop1 cuts one strand of supercoiled DNA by a tyrosine 723 residue located in the C-terminal domain, performing a nucleophilic attack on the DNA backbone [3,4]. The result is the breakage of the phosphodiester bond with the enzyme covalently attached to the 3′ end, forming a reversible cleavable complex made up of hTop1 and DNA [5]. Following the nicking, the broken strand rotates around the complementary intact strand by a mechanism called controlled rotation, that leads to the change of the linking number and consequently to the formation of relaxed DNA [6]. This step is followed by the religation of the breaks, restoration of a double stranded DNA, and finally, the release of the enzyme [7,8]. HTop1 is a well-known molecular target for cancer treatment. For instance, a class of enzyme inhibitors trap hTop1 on DNA, when the enzyme forms a nick, creating a stalled cleavable complex that blocks the enzyme on DNA, leading to cell death by producing a DNA damage, due to the prevention of DNA replication and/or of the transcription machinery complex to proceed [9]. This is the case of the most studied hTop1 inhibitor: camptothecin (CPT) [10,11], whose derivatives, topotecan [12] (TPT) and irinotecan, are currently used in the clinic for treatment of ovarian and non-small cell lung cancer, and colorectal cancer, respectively; while both are used to treat pediatric tumors [13,14]. There are other types of hTop1 inhibitors that act with different mechanisms, such as the prevention of DNA binding and/or inhibition of cleavage by hTop1 [2]. Among them, we find benzoxazines [15], methoxylated Δ5,9 fatty acids [16], kakuol [17] and berberine [18]. The former inhibits the binding of the enzyme to the DNA while the latter prevents cleavage.

Natural products (NPs) remain an extensive and excellent source of inspiration for the development of new pharmaceuticals, and most of the drugs currently used in the clinic are NPs that display inhibition towards a specific target [19]. However, compounds, in their natural form, are often not very active and require a chemical modification to be converted into a more potent molecule exhibiting a stronger biological effect. This is the case of silibinin, extracted from *Silybum marianum*, that alone does not show any effect on hTop1, but inhibits the enzyme, in the form of oxidovanadium (IV) complexes [20]. Another example are chalcones-derived thiosemicarbazones that are efficient in inhibiting hTop1 only when coordinated to a copper atom [21].

**Figure 1:**
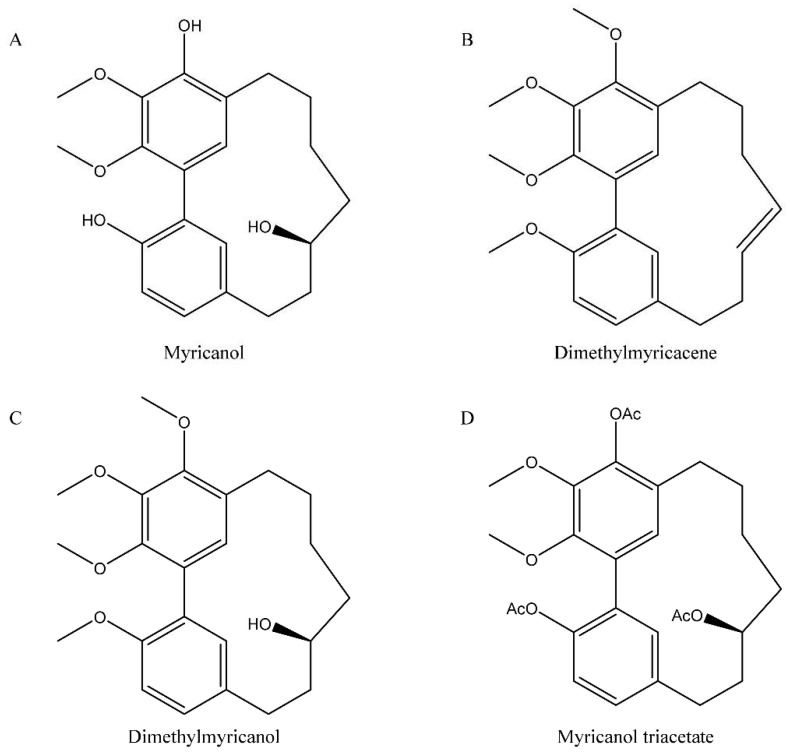
Chemical structure of myricanol and its derivatives. In the figure are reported the structures of myricanol derivatives: (**A**) myricanol, (**B**) dimethylmyricacene, (**C**) dimethylmyricanol and (**D**) myricanol triacetate.

Modification of these naturally occurring compounds led to the discovery of several hTop1 catalytic inhibitors, such as CYB-L10, that has a higher hTop1 inhibition activity, compared with its parent indolizinoquinolinedione [22]. Identification of new hTop1 inhibitors could occur also by virtual screening, where thousands of selected compounds can be docked into the central catalytic domain of the hTop1–DNA complex, as reported by Xin et al. [23]. In line with the strategy of exploring NPs and their derivatives, in this work we report the in vitro and in silico characterization of the action of a synthetic myricanol derivative against hTop1 and the evaluation of its antitumor effect on different cancer cell lines. We tested myricanol and three derivatives, namely dimethylmyricacene (DM), dimethylmyricanol and myricanol triacetate (Figure 1A–D), against hTop1. Among the tested compounds, only DM showed an inhibitory effect against this enzyme. Myricanol is a cyclic diarylheptanoid extracted from *Myrica cerifera* root bark [24]. Reports in the literature show that this compound exhibits several biological activities, including the regulation of the tau protein involved in Alzheimer’s disease, anti-inflammatory and anticancer effects [25]. Concerning its anticancer effect, it has been discovered that myricanol (Figure 1A) can upregulate the expressions of Bax, p21, caspase-3 and caspase-9, and downregulates the expression of Bcl-2, both at the mRNA and protein levels [26]. In this work, we tested myricanol and three derivatives (Figure 1A–D), and found that among them, only DM showed an inhibitory effect against hTop1. Once we determined DM’s inhibitory effect on the enzyme, the compound was further characterized, in terms of its mechanism of action and its potential as an anti-tumor drug against different cancer cell lines.

## 2. Materials and Methods

### 2.1. Protein and Reagents

HTop1, for time course and dose dependent assays, has been expressed in the top1 null EKY3 yeast strain and purified by affinity chromatography, as described elsewhere [27]. To validate the entirety and the activity of the protein, fractions were analyzed by SDS-PAGE, immunoblotting and by a relaxation assay, respectively. For polyacrylamide-based assays, the recombinant htop1 protein (Cat. No. ENZ-306) was purchased from Prospec (Hamada St. 8, Rehovot 7670308, Israel). Topotecan (Hycamtin) was purchased from GlaxoSmithKline (Brentford, Middlesex, TW8 9GS, UK). (3-(4,5-dimethylthiazol-2-yl)-2,5-diphenyltetrazolium bromide (MTT), ACN and DMSO were purchased from Merck (Darmstadt, Germany). DNA oligonucleotides CL14-FITC (5′-GAAAAAAFITCGACTTAG-3′), labelled with fluorescein isothiocyanate (FITC) at the 5′ end, CP25-P phosphorylated at the 5′ end (5′-TAAAAATTTTTCTAAGTCTTTTTTC-3′) and R-11 (5′-GAAAAAATTTT), were purchased from Eurofins Genomics (Sportparkstrasse 2, 85560 Ebersberg, Germany). All compounds are >95% pure through a HPLC analysis.

### 2.2. Cells and Culture Conditions

Dulbecco’s modified Eagle’s medium high glucose (DMEM), RPMI 1640 medium, fetal bovine serum (FBS), L-glutamine and penicillin/streptomycin, were purchased from Euroclone (Pero, Italy), McCoy’s and Minimum essential Medium Eagle (EMEM) were purchased from the Lonza group Ltd. (Basel, Switzerland). Complete media (CM) were supplemented with 10% FBS, 2 mM L-glutamine, 0.1 mg/mL streptomycin and 100 U/mL penicillin. The ovarian cancer cell line SKOV-3 was purchased from Cell Biolabs Inc., and maintained in DMEM-high glucose, CM.

The colorectal adenocarcinoma cell line, HT-29, colorectal carcinoma cell line HCT-116 and melanoma cell SK-MEL-28, were gifted by Dr Cinzia Tesauro (Department of Molecular Biology and Genetics, Aarhus University, 8000 Aarhus C, Denmark) and maintained in RPMI 1640 CM. A non-small-cell lung cancer cell line, A-549, triple negative breast cancer cell SUM-159, MDA-MB-468 and MDA-MB-231, HER2/c-erb-2 positive breast cancer cell line SK-BR-3, MCF-7 and T-47-D ER+, PR+/–, HER2– and the adenocarcinoma HeLa cell line were provided by Dr. Giuseppe Sconocchia (Institute of Translational Pharmacology, National Research Council, CNR) and were maintained in DMEM-high glucose, CM. The submandibular gland squamous cell carcinoma cell line A-253 and hypopharyngeal squamous cell carcinoma cell line FaDu were gently provided by Dr. Angelo Peschiaroli (Institute of Translational Pharmacology, National Research Council, CNR) and maintained in McCoy’s CM and E-MEM CM, respectively. All cell lines were kept in culture for a maximum of eight passages and were tested for mycoplasma, using the PCR detection Kit (Euroclone).

### 2.3. Production and Characterization of DM

DM was produced, according to reference [28]. Briefly, sodium hydride (2 eq) was added over 30 min to a solution of myricacene (10 mg, 0.03 mM) and methyl iodide (5 eq) in 5 mL of dimethylacetamide at 0 °C. The reaction mixture was kept for 4 h at 0 °C then diluted with water and extracted with diethyl ether. The ether extract was washed with water and brine, and was dried over sodium sulfate, filtered and evaporated. The resulting residue was purified by chromatography using normal phase HPLC (Shimadzu LC-20AR system equipped with a Shimadzu SPD-20A UV-vis detector using semipreparative silica conditions) using 25% ethyl acetate and 75% hexane to give 5 mg (0.014 mM, 45% yield) of DM. The ^1^H NMR spectra of DM was recorded in CDCl_3,_ using a Varian 600 MHz broadband instrument and is reported in Supporting Information Figure S3. ADMET predictions were performed using the ADMETLAB2.0 [29] and added in the Supporting Information (Table S1).

### 2.4. Dose Dependent and Time Course Assays

To assess the minimal dose of DM inhibiting hTop1 a dose dependent relaxation assay of negatively supercoiled DNA pBlueScript KSII (-) was performed. In a volume of 30 μL, were mixed TOPO mix buffer 1X (20 mM Tris-HCl pH 7.5, 0.1 mM EDTA, 10 mM MgCl_2_ and 5 μg/mL acetylated bovine serum albumin) with 150 mM KCl, 0.5 μg pBlueScript and 1 U of purified htop1, with different concentrations of DM. As a positive control, the enzyme was incubated with the same amount of ACN used to dissolve the compound. Following 1 h incubation at 37 °C, the reaction was stopped by adding 0.5% SDS stop dye. To evaluate the reversibility or irreversibility of DM, the same experiment was carried out with 100 μM of DM as a function of time, and reactions were stopped at the indicated time points with a final concentration of SDS 0.5%. All samples were run on 1% agarose gel with TBE 1 X buffer (48 mM Tris, 45.5 mM boric acid, 1 mM EDTA). The presence of relaxed supercoiled DNA was visualized through a UV transilluminator after a gel staining in 0.5 μg/mL EtBr and destaining in dH_2_O.

### 2.5. Cleavage and Religation Kinetics

To analyze the cleavage kinetics, the CL14-FITC oligonucleotide was annealed to a CP25-P complementary strand to assemble both cleavage and religation substrates, hereafter indicated as suicide substrate (SS). The cleavage reaction was performed at different time points with 100 μM of DM, by incubating 0.6 pmol of SS with 1.2 pmol hTop1 (Prospec), as previously described [30]. The reactions were stopped by adding 0.5% SDS. The samples were resolved by electrophoresis on a denaturing polyacrylamide gel with 7 M urea and 20% acrylamide 29:1. The gels were visualized, using a ChemiDoc MP and analyzed by densitometry, using Image J software. The plots represent the mean of three independent experiments.

### 2.6. Cell Viability Assays

All tumor cell lines were seeded in a 96-well plate for 24 h before performing the experiment at 37 °C, 5% CO_2_ with the appropriate medium, to evaluate the cell viability, as previously described [31]. The cells were treated with 100 μM of DM or with the same amount of ACN (positive control), to evaluate any possible side effect of the solvent. The plates were then incubated for 48 h at 37 °C under 5% CO_2_. Following the incubation, the medium was then removed and replaced with 0.5 mg/mL of MTT reagent in a final volume of 200 μL diluted with the medium. The plates were incubated again for 4 h at 37 °C, 5% CO_2_. Prior to measuring the absorbance at 570 nm, the medium was replaced with 100 μL of DMSO and placed on a shaking incubator for 15 min at room temperature, covered from light. The dose dependent assay cells were plated in a 96-well plate the day before the experiment. Then, 24 h later, the medium was removed and replaced with different amounts of DM. In detail, the cells were treated with 12.5, 25, 50 and 100 μM of DM and the same concentrations were used to treat cells with TPT as well, representing the positive cytotoxicity control. The plates were incubated again for 48 h at 37 °C under 5% CO_2_. To evaluate the minimal dose of DM reducing cell viability, the cells were subjected to the MTT reagent, as described above.

### 2.7. Molecular Docking

The crystal structure of the human DNA-bound topoisomerase IB (PDB ID: 1T8I), [32] lacking the N-terminal domain (residues 1–200), has been used as a receptor for the molecular docking simulations. The protein structure from the PDB file exhibited a Ramachandran plot with 502 (97.3%) residues at favorable torsion angles, and a further 12 residues (2.3%) at the allowed and 2 (0.4%) at the outlier regions (Figure S4). The CPT molecule present in the crystal structure was removed from the complex [33]. The structure of DM has been drawn using MarvinSketch version 21.17.0, ChemAxon (https://www.chemaxon.com accessed on 22 July 2022) and converted in 3D using the OpenBabel routines [34]. The receptor and drug structure files have been converted into the pdbqt format using the prepare_receptor4.py and prepare_ligand4.py tools of the AutoDockTools4 program [35]. The protein-ligand molecular docking simulations were performed using the AutoDock Vina 1.1.2 program [35]. A molecular docking simulation, including ten docking runs, was performed using a box of size x = 20.25 Å; y = 22.15 Å; z = 21.35 Å, centered over the hTop1-DNA binding site. To increase the accuracy of the binding pose estimation, 10 receptor residue side chains around the binding site were considered as flexible (Arg364, Arg488, Lys532, Asp533, Ile535, Arg590, Asn631, His632, Thr718 and PTyr723). The interaction analyses on the best binding pose was performed using the Maestro software (Schrödinger Release 2022-2: Maestro, Schrödinger, LLC, New York, NY, USA, 2021).

### 2.8. Classical Molecular Dynamics Simulations

The tleap module of the AmberTools19 program [36] was used to generate the topology and coordinates files of the two best complexes obtained from the molecular docking simulations and for the ligand-free hTop1-DNA structure (PDB ID: 1T8I), [36] simulated as a reference. The AMBER ff19SB [36] and parmbsc1 [37] force fields have been used to parametrize the protein and DNA molecules, respectively, while DM parameters were generated using the antechamber module of the AmberTools19 program and the general Amber force field [38]. Each system has been inserted in a triclinic box filled with TIP3P water molecules and neutralized, adding 0.15 mol/L of NaCl, setting a minimum distance of 14.0 Å from the box sides [39]. In order to remove unfavorable interactions, four minimization cycles were performed for the two systems, each composed of 500 steps of the steepest descent minimization, followed by 1500 steps of the conjugated gradient. A starting restraint of 10.0 kcal∙mol^−1^∙Å^−2^ was imposed on the protein, DNA and ligand atoms, being slowly reduced, and finally removed in the last minimization cycle. The system’s temperature was gradually increased from 0 to 300 K in an NVT ensemble, using the Langevin thermostat [40], over a period of 2.0 ns. A starting restraint of 0.5 kcal∙mol^−1^∙Å^−2^ was imposed on the protein, DNA and the ligand atoms, and then gradually decreased to relax the entire system. The systems were then simulated in an isobaric-isothermal (NPT) ensemble for 2.0 ns, using the Langevin barostat, imposing a pressure of 1.0 atm and maintaining the temperature to 300 K [41]. The SHAKE algorithm was used to constrain the bonds involving hydrogen atoms [42]. The pmemd.cuda module of the AMBER16 package has been used to perform 100 ns of production runs for each system, setting a timestep of 2.0 fs [43]. The coordinates have been saved every 1000 steps. The long-range interactions have been treated using the PME method, while a cut-off of 9.0 Å was imposed for short-range interactions [44].

### 2.9. Trajectory Analysis

GROMACS 2021.2 analysis tools [45] have been used to perform the principal component analysis (PCA) on Cα atoms of the hTop1 for the three trajectories [46]. This analysis is based on the diagonalization of a covariance matrix, generated for each trajectory using the covar module of GROMACS, and built from the atomic fluctuations of Cα atoms after the removal of the roto translations. Dynamic cross-correlation maps (DCCMs) were computed, starting from the covariance matrices generated for each trajectory, using in-house written code. Plots were generated using the matplotlib Python3 library [47]. Molecular mechanics/generalized Born and surface area continuum solvation (MM/GBSA) analyses [48] were performed using the MMPBSA.py.MPI program implemented in the AMBER16 software on three nodes of the of ENEA HPC cluster CRESCO6 [49], setting the ionic strength to 0.15 M. The per-residue decomposition analysis was performed on the nucleotides and the residues surrounding the drug molecules.

### 2.10. Statistics

The cleavage and religation kinetics and dose dependent cell viability assay were analyzed by a two-way ANOVA test, using GraphPad Prism. The statistical analysis for the cell viability assay was evaluated by GraphPad Prism software, using the multiple *t*-test. All data were expressed with mean ± SD values. * *p* ≤ 0.05, ** *p* ≤ 0.01, *** *p* ≤ 0.001 and **** *p* ≤ 0.0001.

## 3. Results

### 3.1. DM inhibits the Catalytic Activity of hTop1

**Figure 2:**
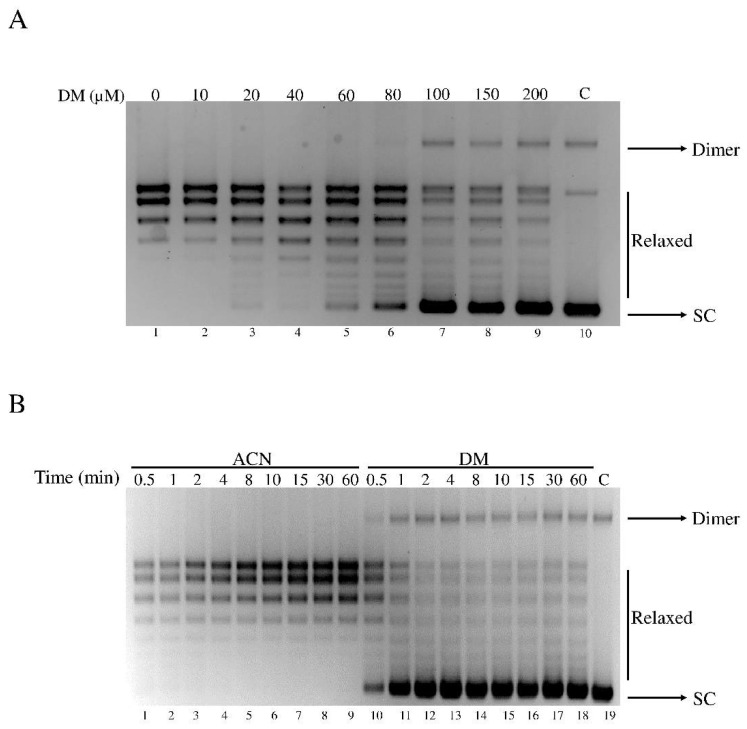
Inhibition of the catalytic activity of hTop1 by DM. (**A**) Relaxation assay in the presence of different concentrations of DM, ranging from 10 µM to 200 µM (lanes 2–9), performed in a dose-dependent manner. Samples were incubated in the presence and absence of DM and incubated for 1 h at 37 °C, then were resolved on an agarose gel and stained with EtBr. Bands were visualized by a UV transilluminator. Lane 0 represents the positive control where the supercoiled DNA is incubated with the solvent and enzyme, lane 10 is the negative control with the supercoiled and DM only. (**B**) Time course assay in the presence of 100 µM DM (lanes 10–19) or with the same amount of ACN (lanes 1–9). The results were analyzed on an agarose gel, stained with EtBr and visualized by a UV transilluminator.

To screen myricanol and its derivatives (Figure 1A–D) against hTop1, we performed a relaxation assay incubating 1 U of the enzyme in the presence and absence of 200 µM of the compounds. Following the 1 h incubation, the samples were analyzed by electrophoresis on agarose gel, to evaluate the presence of supercoiled and/or relaxed DNA. Among all compounds tested, only DM was able to inhibit the hTop1 activity (Supporting Information Figure S1), and therefore we concentrated our attention on DM. To further study the inhibitory effect of DM on hTop1, we performed a plasmid relaxation assay with different concentrations of DM (Figure 2A). HTop1 activity on a supercoiled plasmid, in presence of increasing concentrations of DM, was monitored after 1 h. The results indicate that DM inhibits the relaxation activity of hTop1 in a dose dependent manner (Figure 2A).

The addition of DM to the hTop1 and DNA determines an inhibition of the relaxation activity, already detectable at a concentration of 80 μM (Figure 2A, lane 6), and that becomes maximal at 100 μM (Figure 2A, lane 7). DM does not affect the electrophoretic mobility of DNA in absence of hTop1 at 200 μM (Figure 2A, lane 10). Since DM is dissolved in acetonitrile (ACN), we assayed the activity in the presence of an identical amount of ACN without DM, demonstrating that ACN does not affect the relaxation of hTop1 (Figure 2A, lane 1).

The relaxation assay performed as a function of time with DM at a concentration of 100 μM, shows a lasting effect over time (Figure 2B, lanes 10–18), indicating that the inhibition of the enzyme catalytic activity is irreversible, at least in the time range explored here, up to 1 h. The relaxation assay, adding only ACN, shows that this solvent has no relevant inhibitory effect (Figure 2B, lanes 1–9).

### 3.2. Cleavage Assays in the Absence and Presence of DM

**Figure 3:**
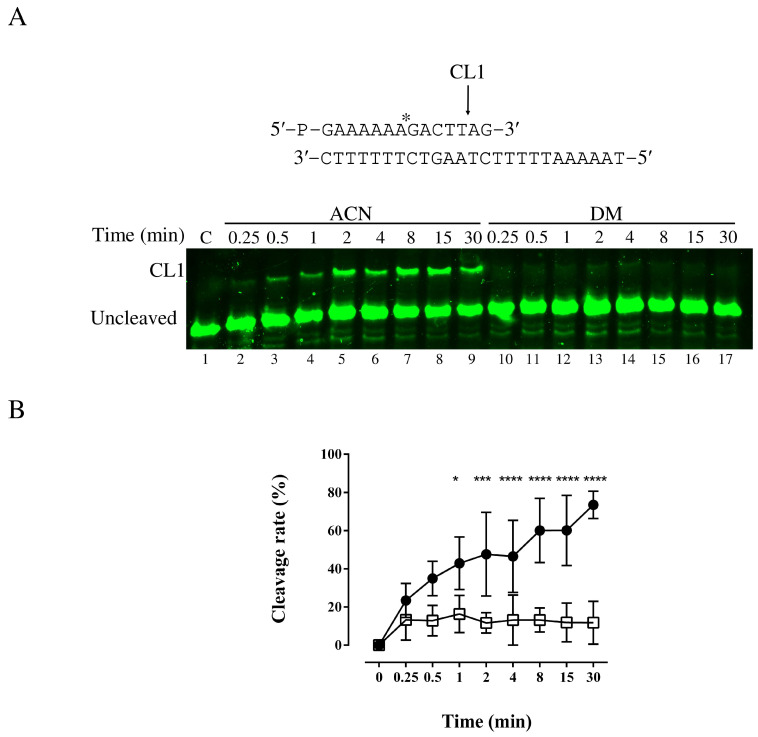
Analysis of Cleavage of the hTop1 catalytic mechanism on the fluorescently labeled SS. (**A**) Cleavage assay of hTop1 in the absence and presence of DM. Top panel is a schematic representation of SS, the bottom panel is a denaturing polyacrylamide gel. Samples were incubated at 25 °C in the absence and presence of 100 µM and the reaction was stopped at different time points with 2.5% SDS. Samples were resolved on a denaturing polyacrylamide gel and visualized by ChemiDoc MP imaging system. CL1 cleaved strand, C is the negative control. (**B**) Plot of the percentage of the cleavage rate as function of time. Black circles represent the enzyme incubated in the presence of ACN while the white squares reported the effect of DM at 100 µM. The plot reports the cumulative data of four independent experiments with mean ± SD values. Asterisks indicate * *p* ≤ 0.05, *** *p* ≤ 0.001, **** *p* ≤ 0.0001.

To characterize which step of the hTop1 catalytic mechanism is affected by DM, the enzyme activity was evaluated on a suicide substrate (SS) in the absence and presence of DM, in a time course experiment, as in [30]. Briefly, the experiment was performed with a fluorescently labelled SS made by the CL14-FITC annealed to the CP25-P oligonucleotide phosphorylated at the 5′ end, to produce a duplex with a 5′ single-strand overhang (Figure 3A). The enzyme action on this substrate generates a suicide product, since the cleaved AG-3′ dinucleotide is too short to be religated, so it can diffuse away leaving the enzyme covalently attached to the oligonucleotide 3′-end. The enzyme (1.2 pmol) was incubated with 100 µM of DM and the reaction was stopped at time points from 0.25 to 30 min, precipitating the samples in 100% ethanol and digested with trypsin. The products were resolved on a denaturing urea polyacrylamide gel. As indicated in Figure 3A lanes 10–17, the cleavage is inhibited by the compound while in presence of only ACN (lanes 2–9), the enzyme activity is not perturbed, as indicated by the percentage of the cleaved fragment (CL1) against time (Figure 3B).

### 3.3. Religation Kinetics

**Figure 4:**
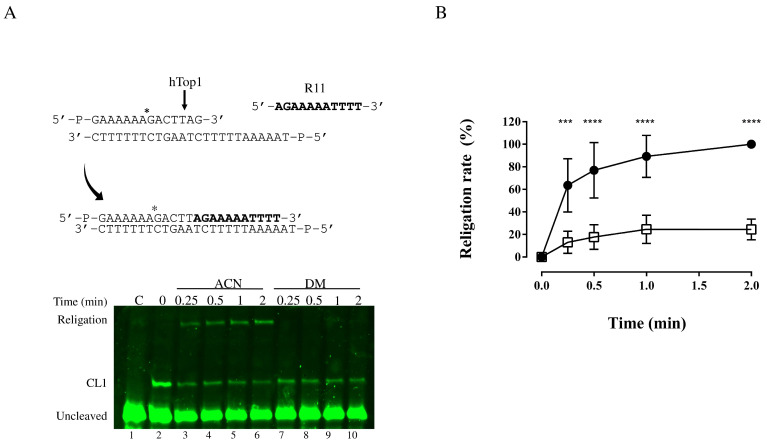
Analysis of the religation of the hTop1 catalytic mechanism on the fluorescently labeled SS. (**A**) Top panel reported the sequences of the fluorescently labeled SS for the religation assay. Bottom panel is a denaturing polyacrylamide gel of the religation assay. Samples were incubated 1 h at 25 °C and then 30 min at 37 °C. Reaction started by adding a 200-fold excess of R11 oligonucleotide in the absence and presence of 100 µM of DM, and then stopped at different time points with 2.5% SDS. CL1 is the cleaved strand, C is the negative control and 0 is the starting condition before adding R11. (**B**) Plot of the religation assay where the percentage of the appearance of the religated bands as function of time is reported. Black circles and white squares represent DM and can, respectively. The figure shows the cumulative data, with mean ± SD values, of three different experiments. Asterisks indicate * *p* ≤ 0.05, *** *p* ≤ 0.001 and **** *p* ≤ 0.0001.

Analysis of the religation step was carried out by incubating 0.6 pmol of the enzyme with 1.2 pmol of the suicide substrate to obtain the cleavage complex (Figure 4A, lane 2). Subsequently, the reaction was divided into two tubes to which we separately added 100 µM of the compound in ACN or ACN alone. The 200-fold molar excess of the complementary R11 oligonucleotide (5′-AGAAAAATTTT-3′) was subsequently added to promote the religation step. The aliquots were removed at different time points, from 0.25 min to 2 min, and the reactions were stopped by adding 2.5% SDS. The reaction products were analyzed by denaturing PAGE (Figure 4A).

The results indicate that the presence of DM strongly inhibits the religation step of the enzyme catalytic cycle (Figure 4A, lanes 7–10), whereas, in the presence of ACN (Figure 4A, lanes 3–6) the enzyme can religate the R11 oligo correctly. The plot of the percentage of the religated product is reported in Figure 4B.

### 3.4. Cell Viability Assay and Dose Dependent Effect

**Figure 5:**
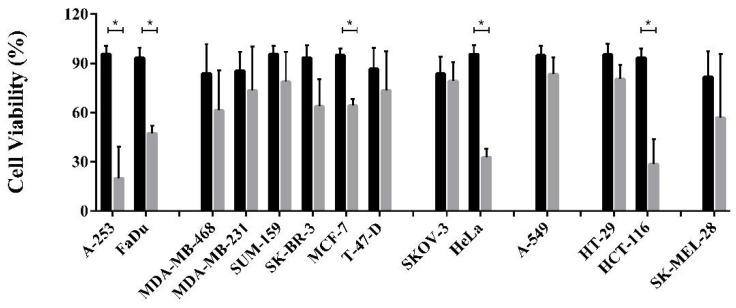
Cell viability assay. Reported cancer cell lines were incubated for 48 h in the presence of ACN (black bars) and 100 µM DM (gray bars) in an incubator at 37 °C, 5% CO_2_. Following the 48 h, the cells were stained with MTT and incubated for 4 h. Cell viability was recorded by measuring the OD at 590 nm. The figure shows the cumulative data of three independent experiments with mean ± SD value. Asterisk indicates * *p* ≤ 0.05.

We next asked whether DM could be used as a possible lead compound to treat cancer cells. To validate the activity of the compound, we performed a cell viability assay on A-253, FaDu, MDA-MB-468, MDA-MB-231, SUM-159, SK-BR-3, T-47-D, SKOV-3, HeLa, A-549, HT-29, HCT-116 and SK-MEL-28 cell lines. In this assay, we observed, as reported in Figure 5, that among all the cell lines tested A-253, FaDu, MCF-7, HeLa and HCT-116 show a significant viability reduction after the 48 h incubation at 37 °C, in the presence of 100 µM DM.

**Figure 6:**
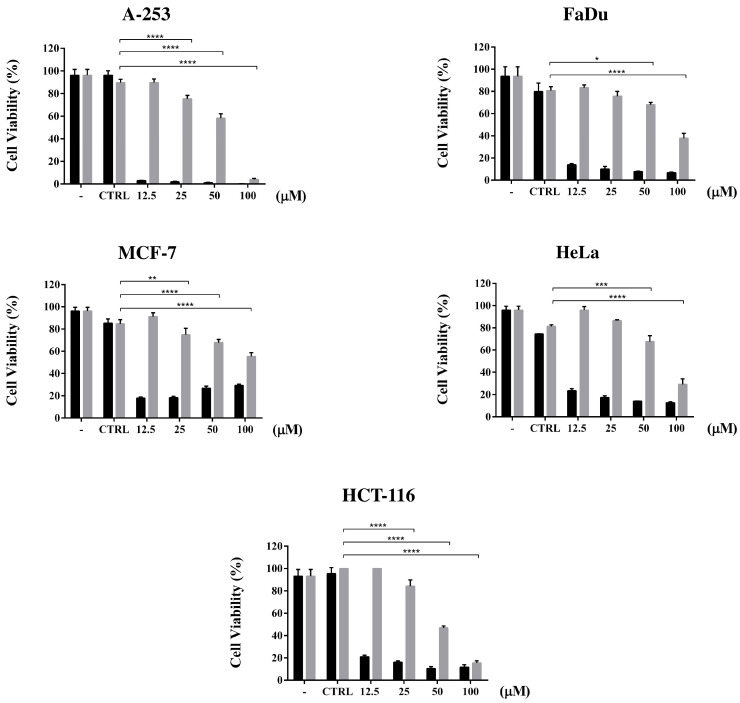
Dose dependent cell viability assay. The figure shows a dose dependent viability in the presence of different concentrations of DM, ranging from 12.5 to 100 µM (gray bars). As positive cytotoxicity control, the cells were incubated with TPT (black bar), as well for 48 h at 37 °C, 5% CO_2_. (-) is the positive control while CTRL represents the cells incubated with the appropriate drugs solvent, DMSO and ACN for TPT and DM, respectively. The reported data represent cumulative experiments with mean ± SD values. Asterisks indicate * *p* ≤ 0.05, ** *p* ≤ 0.01, *** *p* ≤ 0.001 and **** *p* ≤ 0.0001.

Then, to identify the minimal dose that impairs cancer cells viability, the same assay was performed in a dose dependent manner (Figure 6). All of the cell lines significantly affected by DM in the previous experiment were incubated at 37 °C for 48 h with different concentrations of DM, ranging from 12.5 to 100 µM. Viable cells were stained by incubating with the MTT reagent for 4 h. TPT was used as the positive cytotoxicity control (Figure 6 black bars). The percentage of the cell viability reduction was calculated, relative to the control with the solvent DMSO and ACN for TPT and the compound, respectively.

### 3.5. Molecular Docking Simulations

**Figure 7:**
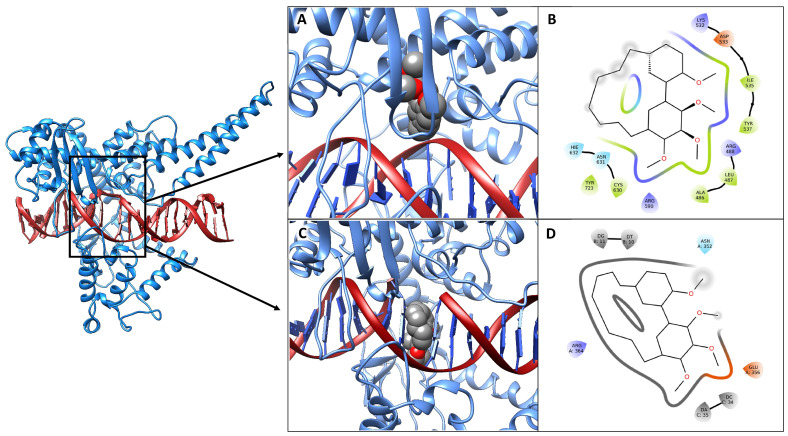
Three-dimensional structure of the hTopI-DNA-drug complexes. The hTop1 structure is represented as a blue cartoon, while the DNA is shown as red ribbons. (**A**) Three-dimensional best binding pose obtained for the drug within the catalytic site. (**C**) Three-dimensional best binding pose obtained for the drug in an intercalated configuration. DM is shown as a space fill model, colored by the atom type, with carbon atoms in grey. The pictures were obtained using Chimera [50]. (**B**,**D**) Two-dimensional schematic view of the interactions between the drug and the hTop1-DNA complex for the two poses. Labels indicate base or residue names and their numbering in the structure. The pictures were created using the Maestro software (Schrödinger Release 2022-2: Maestro, Schrödinger, LLC, New York, NY, USA, 2021).

The interaction of DM with a covalent-bound DNA-bound hTop1 structure has been evaluated, using molecular docking simulations. The system recalls the experimental conditions of incubation of the enzyme, drug and DNA. The docking results indicate that the drug can bind within the hTop1 catalytic pentad or in an intercalated configuration (Figure 7A,C), interacting with the hTop1-DNA complex with an energy of about −9.2 and −9.1 kcal/mol, respectively. Figure 7B–D describes the interaction pattern evaluated for these poses. In the first one, the compound interacts with several protein residues through polar and non-polar interactions, contacting all the residues of the catalytic pentad (Figure 7B, Arg488, Lys532, Arg590, His632 and Tyr723). In the intercalated configuration, the binding pose is characterized by polar and non-polar interactions with Glu356, Arg364, Asn352 and four hydrophobic interactions with the nucleotides among which the molecule is intercalated (Figure 7D).

### 3.6. MM/GBSA Analysis

The best complexes obtained with the molecular docking have been subjected to a 100 ns-long classical molecular dynamics (MD) simulation, to both validate the stability of the complexes and to correctly estimate the interaction energy between the hTop1-DNA and the drug. The results of the MM/GBSA calculations corroborated the strong interaction between DM and the hTop1-DNA structure, which is characterized by an interaction of free energy of −32.6 and −22.7 kcal/mol, for the drug inside the catalytic site or intercalated, respectively (Table 1).

Both vdW and electrostatic interactions contribute to the binding, although the electrostatic contribution is higher when the drug binds within the catalytic pentad. In order to provide an estimate of the contribution given by single residues or DNA bases to the total binding energy of the drug, the MM/GBSA per-residue decomposition analyses (Table 2) were carried out for both the simulations. The analysis confirmed that in the first binding pose, the drug closely interacts with the hTop1 catalytic site residues and also with one of the underlying DNA bases (Table 2, left), while the second pose showed that the drug not only closely interacts with the DNA bases, but that it also establishes interactions with several protein residues located within the loops forming the hTop1 “lips” region (Table 2, right) which are involved in the opening and closing of the protein clamp during the DNA binding and release [2].

Visual inspection of the trajectory indicates a partial rearrangement of DM within the binding site, which turns into a partially intercalated conformation (Figure S2), thus being able to interact with the amino acids of the protein. Moreover, the high interaction free energy and the binding modes of the molecule, which can alter the interactions between DNA and the protein, suggest that DM could interfere with the controlled rotation mechanism that leads to the final DNA relaxation and release from hTop1 [2].

### 3.7. Principal Component and RMSF Analyses

**Figure 8:**
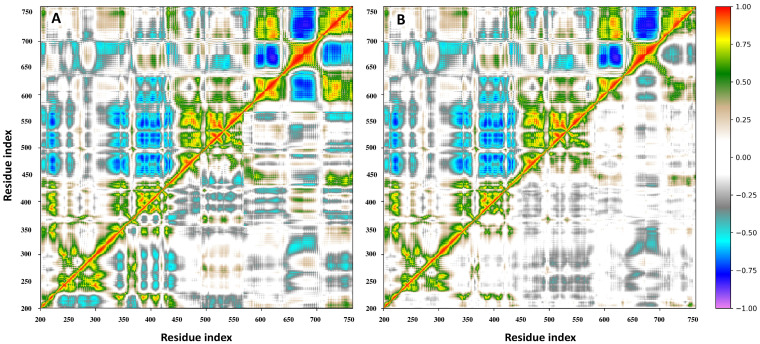
DCCMs obtained for the three simulated systems. The upper-left triangles of the graph represent the DCCM for the unbound hTop1-DNA complex, while the lower-right triangles show the DCCMs for the drug in the catalytic site (**A**) or intercalated within the DNA bases (**B**), respectively. Color coding is reported in the legend. Positive values between two residues indicate a correlated motion, while the negative values indicate an anti-correlated motion.

Dynamic cross-correlation maps (DCCM), based on the atomic fluctuations of 565 Cα atoms of hTop1, were generated from the three MD trajectories of the hTop1-DNA and hTop1-DNA-drug complexes, to evaluate whether the presence of the drug could induce changes in the structural-dynamical properties of the enzyme. In this representation, the positive values indicate a correlation of the motion between the two residues, which move in the same direction in space, while negative ones represent an anti-correlated motion, with residues moving in opposite directions. Indeed, MD simulations identify strong differences between the unbound and bound complexes and show the presence of functional structural motions in the hTop1 unbound structure (Figure 8A,B, upper left triangles).

**Figure 9:**
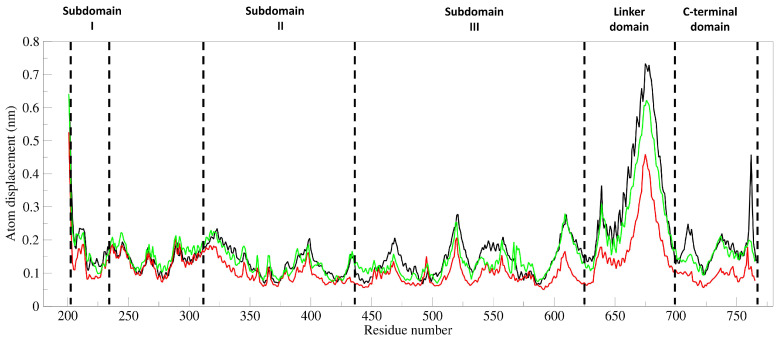
Atomic displacement. Atomic displacement calculated for each of the 565 C-alpha atoms of hTop1, computed along the direction of the first eigenvector for the three MD trajectories. The black line indicates the unbound hTop1-DNA complex, while the red and green lines indicate the complex with DM in the catalytic site or intercalated within the DNA bases, respectively. Dotted lines separate the DNA region from the different hTop1 domains, as indicated by the labels.

The unbound hTop1-DNA simulation is characterized by the presence of highly correlated and anti-correlated motions, that underpin the catalytical mechanism, as already described in our previous studies. The presence of the DM within the binding pocket almost completely destroys these correlated motions (Figure 8A,B, lower right triangles). The presence of the compound causes the intra- and inter-domain protein motions to become almost completely uncorrelated, except for a small anti-correlated region between subdomain I (residues 300–350) and the linker region (residues 636–712) and the scattered positively correlated motions in proximity of the three subdomains of the hTop1 core region (residues 250–575). To characterize the main regions showing different flexibilities in the bound and unbound hTop1-DNA complexes, we performed a principal component analysis (PCA) on the two MD trajectories. This technique identifies the principal 3N directions defining most of the protein motion. Atomic displacements, calculated for each Cα atom of hTop1 along the first eigenvector, show that the unbound hTop1 structure has globally a higher degree of flexibility, mainly located in the linker domain, in subdomain III, and in the C-terminal domain (Figure 9, black line). The linker is the most flexible region of hTop1 in both the systems, but in the unbound one, it is characterized by about two-fold the flexibility observed in the bound states (Figure 9, red and green lines).

These results confirm the presence of highly correlated motions between the three principal hTop1 domains in the unbound structure (Figure 8A, B, upper-left triangles), and further corroborate the hypothesis that the presence of DM induces an increase in the rigidity of the entire enzyme.

## 4. Discussion and Conclusions

Nature is the main source of compounds that, with appropriate chemical modifications, could be used to develop more efficient drugs to treat diseases, such as cancer. Most of the anticancer drugs that are currently used in the clinic are derived from NPs that, with the appropriate modification, could lead to the formulation of more efficient drugs. This is the case of CPT and its derivatives: TPT and irinotecan, both inhibiting the religation reaction of the essential protein hTop1. Another example is the indolizinoquinolinedione derivative CYB-L10 that inhibits the hTop1 catalytic cleavage reaction and prevents the formation of a cleavable complex. In addition to this mode of action, a drug can inhibit also both reactions, as is the case of erybraedin C (Ery-C). In the work of Tesauro et al., it was demonstrated that Ery-C is an in vitro catalytic inhibitor of hTop1, that efficiently acts on both the cleavage and the religation reactions [51]. DM, a semisynthetic diarylheptanoid derived from myricanol extracted from the bark of *Myrica cerifera* exhibits the same behavior of Ery-C, (Figure 3 and Figure 4): this drug inhibits the hTop1 cleavage and religation. We characterized the system with orthogonal techniques and give a clearer view of the mechanism actions of these compounds, from the nanosecond-nanometer range to hours-centimeter, with biochemical, structural and atomistic details as detectable by the techniques used (PAGE, step specific functional assays, modeling, MD simulations, Docking runs, etc.). DM inhibition is also endowed with a seemingly irreversible behavior, we can speculate because of the two free energy basins associated with the different binding conformations, in which the complex may be kinetically trapped. Although this is generally not a desired property in a lead compound, the chemical modifications could allow to develop a reversible behavior. The reported data demonstrate that DM certainly targets hTop1, as reported by the agarose-based assay and corroborated by the energetic analysis of poses in the computational studies, but it is not, probably the unique target.

The inhibitory effect of the drug on different cancer cell lines, reported in Figure 5 and Figure 6, shows an impact that is quite smaller than TPT and more surprisingly, uncorrelated with the expression levels of hTop1. Indeed, the cell lines that do not express extremely high levels of hTop1, show a viability reduction as much as those which express a higher amount of enzyme. Thus, the tested compound could have additional cellular targets besides hTop1. This multitarget mechanism of action could also be hinted by the dramatic reduction of the viability inhibition by the drug as soon as the concentration decreases. Nevertheless, its activity on hTop1 is quite efficient, as it uncouples the concerted motions typically exhibited by the working enzyme (Figure 8) [52].

Further experiments need to be performed to clarify the off-target mechanism, and additional chemical modifications need to be explored to improve the DM activity against hTop1, in order to develop a new anticancer drug targeting only hTop1, that could be used in clinic.

## Figures and Tables

**Table 1:** Results of the MM/GBSA analyses of the MD trajectories of the hTop1-DNA complex with the drug.

Binding Site	vdW * (kcal/mol)	Electrostatic (kcal/mol)	Interaction Energy (kcal/mol)
Catalytic site	−42.7	−17.9	−32.6
DNA-intercalated	−42.3	−9.5	−22.7

* van der Waals.

**Table 2:** This MM/GBSA per-nucleotide/residue decomposition analyses.

Catalytic Site ^1^		DNA-Intercalated ^1^	
Residue/Nucleotide	Binding Energy (kcal/mol)	Residue/Nucleotide	Binding Energy (kcal/mol)
Ala486	−2.35	Ala351	−1.15
Leu487	−3.60	Asn352	−3.49
Arg488	−11.93	Glu356	−1.72
Lys532	−1.89	Arg364	−0.92
Asp533	−0.93	Lys374	−0.50
Tyr537	−3.10	Trp416	−0.71
Arg590	−2.94	Lys425	−3.41
Cys630	−2.14	Tyr426	−0.47
Asn631	−5.84	Met428	−0.69
His632	−1.82	dT10	−1.96
Tyr723	−1.25	dG12	−0.30
dG 11	−1.92	dC34	−7.30
		dA35	−6.13

^1^ Analysis was performed for the MD trajectory of the hTop1-DNA complexes bound to DM. Interaction energies were evaluated between the drug and surrounding DNA bases or hTop1 residues.

## Supplementary Materials

The following supporting information can be downloaded at: https://www.mdpi.com/article/10.3390/cells11213486/s1, Figure S1: Inhibition of the catalytic activity of hTop1 by myricanol and its derivatives; Figure S2: 3D structure of the hTop1-DNA in complex with the intercalated drug after 50 ns of the MD simulation time; Figure S3: ^1^H NMR spectrum of dimethylmyricacene (600 MHz, CDCl3 on a Varian 600 MHz broadband instrument); Figure S4: Ramachandran graph for 1T8I crystal structure: green crosses on the grey regions mean favourable torsion angles (502 residues, 97.3%), orange filled triangles on the border regions means allowed torsion angles (12 residues, 2.3%), while red filled circles mean outlier side chain torsion (2 residues, 0.4%); Table S1: In silico ADMET properties for DM as predicted by ADMETlab2.0 server.

## Abbreviations

hTop1, human DNA topoisomerase 1B; MTT, 3-(4,5-Dimethyl-2-thiazolyl)-2,5-diphenyl-2H-tetrazolium bromide; ACN, acetonitrile; EtBr, ethidium bromide; CPT, camptothecin; TPT, topotecan; Ery-C, erybraedin C; DCCM, Dynamic cross-correlation maps; PCA, principal component analysis; FITC, fluorescein isothiocyanate; MD, molecular dynamics.

## References

[1] Stewart L., Ireton G.C., Champoux J.J. (1996). The domain organization of human topoisomerase I. J. Biol. Chem..

[2] Soren B.C., Dasari J.B., Ottaviani A., Lacovelli F., Fiorani P. (2019). Topoisomerase IB: A relaxing enzyme for stressed DNA. Cancer Drug Resist.

[3] Champoux J.J. (2001). DNA topoisomerases: Structure, function, and mechanism. Annu. Rev. Biochem..

[4] Champoux J.J. (1998). Domains of human topoisomerase I and associated functions. Prog. Nucleic Acid Res. Mol. Biol..

[5] Chillemi G., Fiorani P., Benedetti P., Desideri A. (2003). Protein concerted motions in the DNA–human topoisomerase I complex. Nucleic Acids Res..

[6] Chillemi G., Fiorani P., Castelli S., Bruselles A., Benedetti P., Desideri A. (2005). Effect on DNA relaxation of the single Thr718Ala mutation in human topoisomerase I: A functional and molecular dynamics study. Nucleic Acids Res..

[7] Krogh B.O., Shuman S. (2000). Catalytic mechanism of DNA topoisomerase IB. Mol. Cell.

[8] Stewart L. (1998). A Model for the Mechanism of Human Topoisomerase I. Science.

[9] Vos S.M., Tretter E.M., Schmidt B.H., Berger J.M. (2011). All tangled up: How cells direct, manage and exploit topoisomerase function. Nat. Rev. Mol. Cell Biol..

[10] Li F., Jiang T., Li Q., Ling X. (2017). Camptothecin (CPT) and its derivatives are known to target topoisomerase I (Top1) as their mechanism of action: Did we miss something in CPT analogue molecular targets for treating human disease such as cancer?. Am. J. Cancer Res..

[11] Rasheed Z.A., Rubin E.H. (2003). Mechanisms of resistance to topoisomerase I-targeting drugs. Oncogene.

[12] Mancini G., D’Annessa I., Coletta A., Sanna N., Chillemi G., Desideri A. (2010). Structural and dynamical effects induced by the anticancer drug topotecan on the human topoisomerase I–DNA complex. PLoS ONE.

[13] Pommier Y., Pourquier P., Urasaki Y., Wu J., Laco G.S. (1999). Topoisomerase I inhibitors: Selectivity and cellular resistance. Drug Resist. Updat..

[14] Sooryakumar D., Dexheimer T.S., Teicher B.A., Pommier Y. (2011). Molecular and Cellular Pharmacology of the Novel Noncamptothecin Topoisomerase I Inhibitor Genz-644282. Mol. Cancer Ther..

[15] Foto E., Özen Ç., Zilifdar F., Tekiner-Gülbaş B., Yıldız İ., Akı-Yalçın E., Diril N., Yalçın İ. (2020). Benzoxazines as new human topoisomerase I inhibitors and potential poisons. DARU J. Pharm. Sci..

[16] Carballeira N.M., Montano N., Amador L.A., Rodríguez A.D., Golovko M.Y., Golovko S.A., Reguera R.M., Álvarez-Velilla R., Balaña-Fouce R. (2016). Novel Very Long-Chain α-Methoxylated Δ5,9 Fatty Acids from the Sponge Asteropus Niger Are Effective Inhibitors of Topoisomerases IB. Lipids.

[17] Castelli S., Vieira S., D’Annessa I., Katkar P., Musso L., Dallavalle S., Desideri A. (2013). A derivative of the natural compound kakuol affects DNA relaxation of topoisomerase IB inhibiting the cleavage reaction. Arch. Biochem. Biophys..

[18] Vieira S., Castelli S., Falconi M., Takarada J., Fiorillo G., Buzzetti F., Lombardi P., Desideri A. (2015). Role of 13-(di)phenylalkyl berberine derivatives in the modulation of the activity of human topoisomerase IB. Int. J. Biol. Macromol..

[19] Dias D.A., Urban S., Roessner U. (2012). A Historical Overview of Natural Products in Drug Discovery. Metabolites.

[20] León I.E., Cadavid-Vargas J.F., Tiscornia I., Porro V., Castelli S., Katkar P., Desideri A., Bollati-Fogolin M., Etcheverry S.B. (2015). Oxidovanadium(IV) complexes with chrysin and silibinin: Anticancer activity and mechanisms of action in a human colon adenocarcinoma model. J. Biol. Inorg. Chem..

[21] Vutey V., Castelli S., D’Annessa I., Sâmia L.B.P., Souza-Fagundes E.M., Beraldo H., Desideri A. (2016). Human topoisomerase IB is a target of a thiosemicarbazone copper(II) complex. Arch. Biochem. Biophys..

[22] Yu Q., Chen Y., Yang H., Zhang H.L., Agama K., Pommier Y., An L.K. (2019). The antitumor activity of CYB-L10, a human topoisomerase IB catalytic inhibitor. J. Enzyme Inhib. Med. Chem..

[23] Xin L.-T., Liu L., Shao C.-L., Yu R.-L., Chen F.-L., Yue S.-J., Wang M., Guo Z.-L., Fan Y.-C., Guan H.-S. (2017). Discovery of DNA Topoisomerase I Inhibitors with Low-Cytotoxicity Based on Virtual Screening from Natural Products. Mar. Drugs.

[24] Jones J.R., Lebar M.D., Jinwal U.K., Abisambra J.F., Koren III J., Blair L., O’Leary J.C., Davey Z., Trotter J., Johnson A.G. (2011). The Diarylheptanoid (+)-aR,11S-Myricanol and Two Flavones from Bayberry. J. Nat. Prod..

[25] Martin M.D., Calcul L., Smith C., Jinwal U.K., Fontaine S.N., Darling A., Seeley K., Wojtas L., Narayan M., Gestwicki J.E. (2015). Synthesis, stereochemical analysis, and derivatization of myricanol provide new probes that promote autophagic Tau clearance. ACS Chem. Biol..

[26] Dai G., Tong Y., Chen X., Ren Z., Ying X., Yang F., Chai K. (2015). Myricanol induces apoptotic cell death and anti-tumor activity in non-small cell lung carcinoma in vivo. Int. J. Mol. Sci..

[27] Zuccaro L., Tesauro C., Cerroni B., Ottaviani A., Knudsen B.R., Balasubramanian K., Desideri A. (2014). Rolling circle amplification-based detection of human topoisomerase I activity on magnetic beads. Anal. Biochem..

[28] Dickey C., Jinwal U., Calcul L., Baker B.J., Lebar M. (2017). Myricanol Derivatives and Uses Thereof for Treatment of Neurodegenerative Diseases. U.S. Patent.

[29] Xiong G., Wu Z., Yi J., Fu L., Yang Z., Hsieh C., Yin M., Zeng X., Wu C., Lu A. (2021). ADMETlab 2.0: An integrated online platform for accurate and comprehensive predictions of ADMET properties. Nucleic Acids Res..

[30] Ottaviani A., Welsch J., Agama K., Pommier Y., Desideri A., Baker B.J., Fiorani P. (2022). From Antarctica to cancer research: A novel human DNA topoisomerase 1B inhibitor from Antarctic sponge Dendrilla antarctica. J. Enzyme Inhib. Med. Chem..

[31] Soren B.C., Dasari J.B., Ottaviani A., Messina B., Andreotti G., Romeo A., Iacovelli F., Falconi M., Desideri A., Fiorani P. (2021). In vitro and in silico characterization of an antimalarial compound with antitumor activity targeting human DNA topoisomerase IB. Int. J. Mol. Sci..

[32] Lue N., Sharma A., Mondragón A., Wang J.C. (1995). A 26 kDa yeast DNA topoisomerase I fragment: Crystallographic structure and mechanistic implications. Structure.

[33] Staker B.L., Feese M.D., Cushman M., Pommier Y., Zembower D., Stewart L., Burgin A.B. (2005). Structures of three classes of anticancer agents bound to the human topoisomerase I-DNA covalent complex. J. Med. Chem..

[34] O’Boyle N.M., Banck M., James C.A., Morley C., Vandermeersch T., Hutchison G.R. (2011). Open Babel: An Open chemical toolbox. J. Cheminform..

[35] Morris G.M., Ruth H., Lindstrom W., Sanner M.F., Belew R.K., Goodsell D.S., Olson A.J. (2009). AutoDock4 and AutoDockTools4: Automated docking with selective receptor flexibility. J. Comput. Chem..

[36] Salomon-Ferrer R., Case D.A., Walker R.C. (2013). An overview of the Amber biomolecular simulation package. Wiley Interdiscip. Rev. Comput. Mol. Sci..

[37] Ivani I., Dans P.D., Noy A., Pérez A., Faustino I., Hospital A., Walther J., Andrio P., Goñi R., Balaceanu A. (2015). Parmbsc1: A refined force field for DNA simulations. Nat. Methods.

[38] Wang J., Wolf R.M., Caldwell J.W., Kollman P.A., Case D.A. (2004). Development and testing of a general amber force field. J. Comput. Chem..

[39] Jorgensen W.L., Chandrasekhar J., Madura J.D., Impey R.W., Klein M.L. (1983). Comparison of simple potential functions for simulating liquid water. J. Chem. Phys..

[40] Goga N., Rzepiela A.J., De Vries A.H., Marrink S.J., Berendsen H.J.C. (2012). Efficient algorithms for langevin and DPD dynamics. J. Chem. Theory Comput..

[41] Sun D.Y., Gong X.G. (2002). A new constant-pressure molecular dynamics method for finite systems. J. Phys. Condens. Matter.

[42] Miyamoto S., Kollman P.A. (1992). SETTLE: An analytical version of the SHAKE and RATTLE algorithm for rigid water models. J. Comput. Chem..

[43] Da Case D.A., Betz R.M.R., Cerutti D.D.S., Te C.I., Darden T.A., Duke R.E., Giese T.J., Gohlke H., Goetz A.W., Homeyer N. (2016). Amber 2016. Univ. California San Fr..

[44] Darden T., York D., Pedersen L. (1993). Particle mesh Ewald: An N·log(N) method for Ewald sums in large systems. J. Chem. Phys..

[45] Abraham M.J., Murtola T., Schulz R., Páll S., Smith J.C., Hess B., Lindah E. (2015). Gromacs: High performance molecular simulations through multi-level parallelism from laptops to supercomputers. SoftwareX.

[46] Amadei A., Linssen A.B.M., Berendsen H.J.C. (1993). Essential dynamics of proteins. Proteins Struct. Funct. Genet..

[47] Hunter J.D. (2007). Matplotlib: A 2D Graphics Environment. Comput. Sci. Eng..

[48] Genheden S., Ryde U. (2015). The MM/PBSA and MM/GBSA methods to estimate ligand-binding affinities. Expert Opin. Drug Discov..

[49] Iannone F., Ambrosino F., Bracco G., De Rosa M., Funel A., Guarnieri G., Migliori S., Palombi F., Ponti G., Santomauro G. (2019). CRESCO ENEA HPC clusters: A working example of a multifabric GPFS Spectrum Scale layout. Proceedings of the 2019 International Conference on High Performance Computing and Simulation, HPCS 2019.

[50] Pettersen E.F., Goddard T.D., Huang C.C., Couch G.S., Greenblatt D.M., Meng E.C., Ferrin T.E. (2004). UCSF Chimera—A visualization system for exploratory research and analysis. J. Comput. Chem..

[51] Tesauro C., Fiorani P., D’Annessa I., Chillemi G., Turchi G., Desideri A. (2010). Erybraedin C, a natural compound from the plant Bituminaria bituminosa, inhibits both the cleavage and religation activities of human topoisomerase I. Biochem. J..

[52] Fiorani P., Tesauro C., Mancini G., Chillemi G., D’Annessa I., Graziani G., Tentori L., Muzi A., Desideri A. (2009). Evidence of the crucial role of the linker domain on the catalytic activity of human topoisomerase I by experimental and simulative characterization of the Lys681Ala mutant. Nucleic Acids Res..

