# Supplementary material for: Dimethylmyricacene: An In Vitro and In Silico Study of a Semisynthetic Non-Camptothecin Derivative Compound, Targeting Human DNA Topoisomerase 1B
Source: Cells. 2022 Nov 3;11(21):3486. doi: 10.3390/cells11213486 (PMC9654473; doi:10.3390/cells11213486)
Supplement: Supplementary file 1 [file cells-11-03486-s001.zip › Supporting_Information_Ottaviani_et_al.pdf]

Supporting Information

Dimethylmyricacene: an *in vitro* and *in silico* study of a semisynthetic non-camptothecin derivative compound, targeting human DNA topoisomerase 1B.

Alessio Ottaviani[a,d]*, Federico Iacovelli[a], Joshua Welsch[b], Blasco Morozzo della Rocca[a], Alessandro Desideri[a], Mattia Falconi[a], Laurent Calcul[b], Bill J. Baker[b] and Paola Fiorani[a,c].

AUTHORS ADDRESSES

[a]Department of Biology, University of Rome Tor Vergata, 00133 Rome, Italy.

[b]Department of Chemistry, University of South Florida, Tampa, FL, USA

[c]Institute of Translational Pharmacology, National Research Council, CNR, 00133 Rome, Italy.

[d] Department of Onco-hematology, Gene and Cell Therapy, Bambino Gesù Children's Hospital-IRCCS, 00146 Rome, Italy.

A.O. present address. Department of Onco-hematology, Gene and Cell Therapy, Bambino Gesù Children's Hospital-IRCCS, Rome, Italy.

*Corresponding author email: alessio.ottaviani@uniroma2.it

TABLE OF CONTENTS:

Figure S1 S2

Figure S2 S3

Figure S3 S4

Figure S4 S5

Table S1 S6

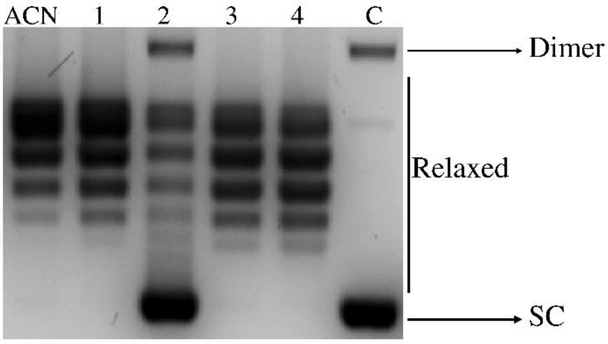

Figure S1. Inhibition of catalytic activity of hTop1 by Myricanol and its derivatives. Relaxation assay in presence of 100 µM of the myricanol **1**, dimethylmyricacene **2**, dimethylmyricanol **3** and myricanol triacetate **4**. Samples were incubated for 1 h at 37°C, resolved on an agarose gel, stained with EtBr and the bands were visualized by UV transilluminator. ACN, represent the positive control where supercoiled DNA is incubated with solvent and enzyme; C, is the negative control with supercoiled DNA only. SC supercoiled.

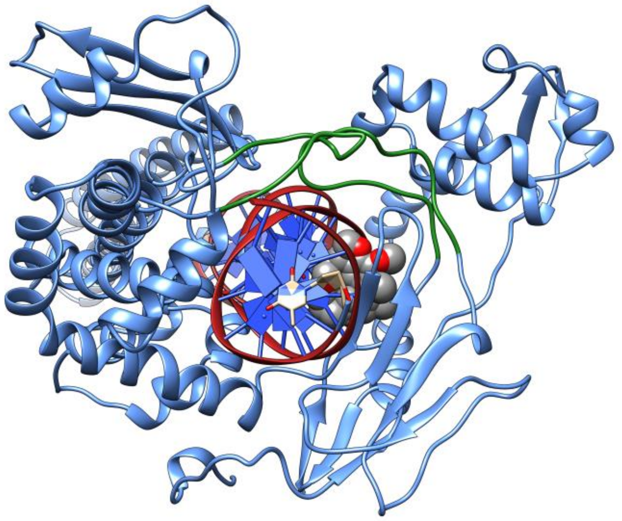

Figure S2. 3D structure of the hTop1-DNA in complex with the intercalated drug after 50 ns of MD simulation time. The hTop1 structure is represented as a blue cartoon, the DNA is shown as red ribbons, the drug as spheres. The analysis of this configuration suggests a partial rearrangement of the **2** within the binding site, allowing its interaction with the loops composing the “lips” of the hTop1, highlighted by the green color.

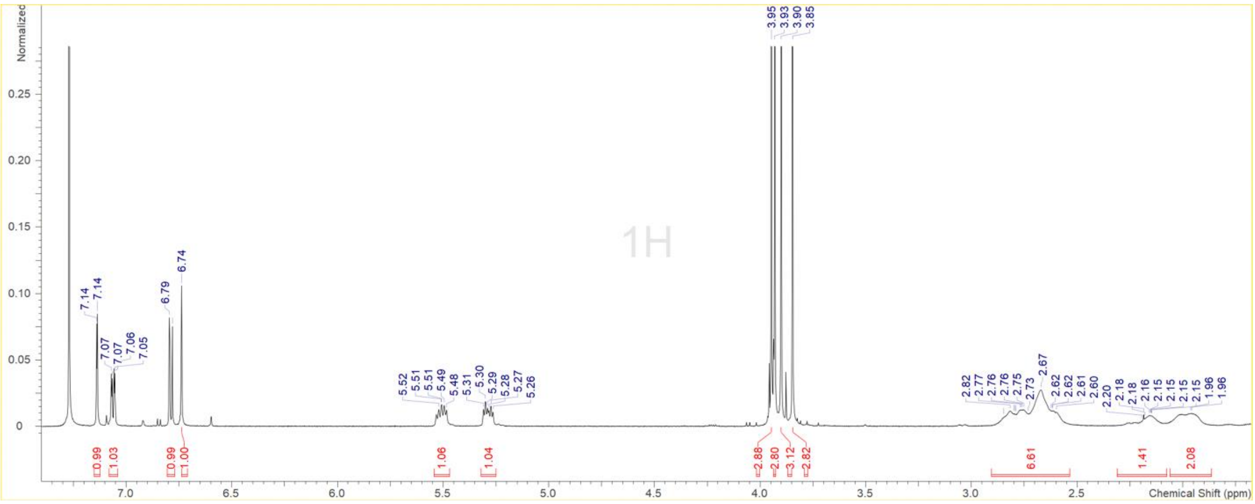

Figure S3. ^{1}H NMR spectrum of dimethylmyricacene (600 MHz, $CDCl_3$ on a Varian 600 MHz broadband instrument)

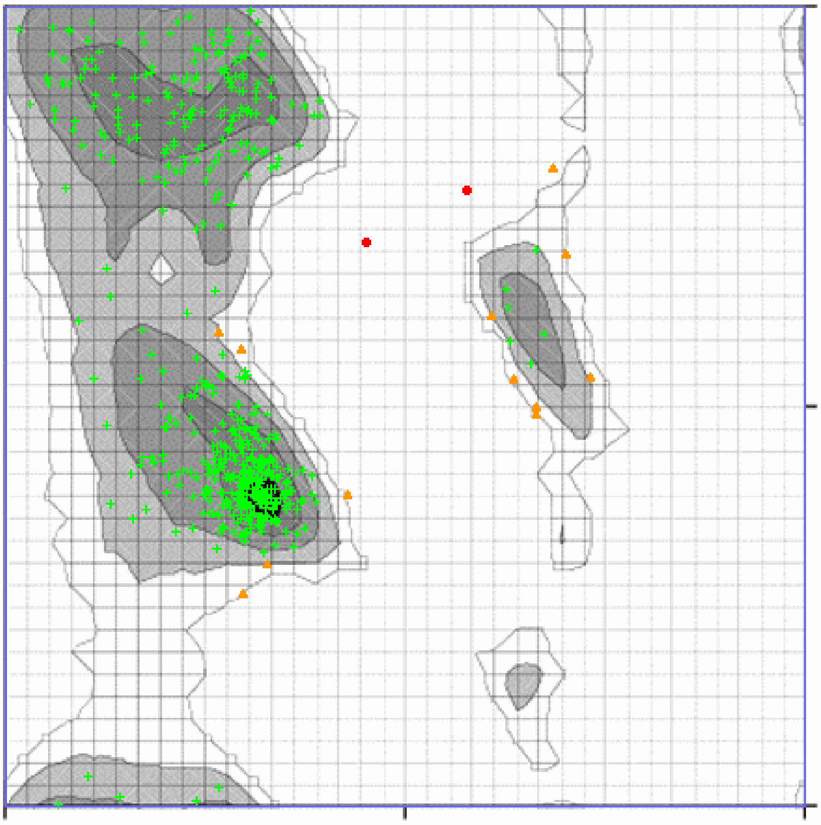

Figure S4. Ramachandran graph for 1T8I crystal structure: green crosses on the grey regions mean favourable torsion angles (502 residues, 97.3%), orange filled triangles on the border regions means allowed torsion angles (12 residues, 2.3%), while red filled circles mean outlier side chain torsion (2 residues, 0.4%).

Table S1. *In silico* ADMET properties for DM as predicted by ADMETlab2.0 server (in the following pages)

1. Physicochemical Property

Property	Value	Comment
Molecular Weight	368.2	Contain hydrogen atoms. Optimal:100~600
Volume	397.4	Van der Waals volume
Density	0.927	Density = MW / Volume
nHA	4	Number of hydrogen bond acceptors. Optimal:0~12
nHD	0	Number of hydrogen bond donors. Optimal:0~7
nRot	4	Number of rotatable bonds. Optimal:0~11
nRing	3	Number of rings. Optimal:0~6
MaxRing	17	Number of atoms in the biggest ring. Optimal:0~18
nHet	4	Number of heteroatoms. Optimal:1~15
fChar	0	Formal charge. Optimal:-4 ~4
nRig	21	Number of rigid bonds. Optimal:0~30
Flexibility	0.19	Flexibility = nRot /nRig
Stereo Centers	0	Optimal: ≤ 2
TPSA	36.92	Topological Polar Surface Area. Optimal:0~140
logS	-6.326	Log of the aqueous solubility. Optimal: -4~0.5 log mol/L
logP	5.293	Log of the octanol/water partition coefficient. Optimal: 0~3
logD	4.077	logP at physiological pH 7.4. Optimal: 1~3

2. Medicinal Chemistry

Property	Value	Decision	Comment
QED	0.694	🟢	■ A measure of drug-likeness based on the concept of desirability;<br>■ Attractive: > 0.67; unattractive: 0.49~0.67; too complex: < 0.34
SAscore	4.183	🟢	■ Synthetic accessibility score is designed to estimate ease of synthesis of drug-like molecules.<br>■ SAscore ≥ 6, difficult to synthesize; SAscore <6, easy to synthesize
Fsp3	0.391	🔴	■ The number of sp3 hybridized carbons / total carbon count, correlating with melting point and solubility.<br>■ Fsp^3 ≥0.42 is considered a suitable value.
MCE-18	39.312	🔴	■ MCE-18 stands for medicinal chemistry evolution.<br>■ MCE-18≥45 is considered a suitable value.

ADMETlab 2.0

NPscore	1.046	-	■ Natural product-likeness score.<br>■ This score is typically in the range from –5 to 5. The higher the score is, the higher the probability is that the molecule is a NP.
Lipinski Rule	Accepted	●	■ MW ≤ 500; logP ≤ 5; Hacc ≤ 10; Hdon ≤ 5<br>■ If two properties are out of range, a poor absorption or permeability is possible, one is acceptable.
Pfizer Rule	Rejected	●	logP > 3; TPSA < 75<br>Compounds with a high log P (>3) and low TPSA (<75) are likely to be toxic.
GSK Rule	Rejected	●	■ MW ≤ 400; logP ≤ 4<br>■ Compounds satisfying the GSK rule may have a more favorable ADMET profile
Golden Triangle	Accepted	●	■ 200 ≤ MW ≤ 50; -2 ≤ logD ≤ 5<br>■ Compounds satisfying the Golden Triangle rule may have a more favorable ADMET profile.
PAINS	0 alerts	-	Pan Assay Interference Compounds, frequent hitters, Alpha-screen artifacts and reactive compound.
ALARM NMR	0 alerts	-	Thiol reactive compounds.
BMS	0 alerts	-	Undesirable, reactive compounds.
Chelator Rule	0 alerts	-	Chelating compounds.

3. Absorption

Property	Value	Decision	Comment
Caco-2 Permeability	-4.786	●	Optimal: higher than -5.15 Log unit
MDCK Permeability	1e-05	●	■ low permeability: < 2 × 10^{-6} cm/s<br>■ medium permeability: 2–20 × 10^{-6} cm/s<br>■ high passive permeability: > 20 × 10^{-6} cm/s
Pgp-inhibitor	0.997	●	■ Category 1: Inhibitor; Category 0: Non-inhibitor;<br>■ The output value is the probability of being Pgp-inhibitor
Pgp-substrate	0.083	●	■ Category 1: substrate; Category 0: Non-substrate;<br>■ The output value is the probability of being Pgp-substrate
HIA	0.003	●	■ Human Intestinal Absorption<br>■ Category 1: HIA+(HIA < 30%); Category 0: HIA-(HIA < 30%); The output value is the probability of being HIA+
$F_{20\%}$	0.004	●	■ 20% Bioavailability<br>■ Category 1: $F_{20\%}$+ (bioavailability < 20%); Category 0: $F_{20\%}$- (bioavailability ≥ 20%); The output value is the probability of being $F_{20\%}$+

ADMETlab 2.0

$F_{30\%}$	0.08	🟢	■ 30% Bioavailability<br>■ Category 1: $F_{30\%}$+ (bioavailability < 30%); Category 0: $F_{30\%}$- (bioavailability ≥ 30%); The output value is the probability of being $F_{30\%}$+

4. Distribution

Property	Value	Decision	Comment
PPB	93.57%	🔴	■ Plasma Protein Binding<br>■ Optimal: < 90%. Drugs with high protein-bound may have a low therapeutic index.
VD	0.588	🟢	■ Volume Distribution<br>■ Optimal: 0.04-20L/kg
BBB Penetration	0.028	🟢	■ Blood-Brain Barrier Penetration<br>■ Category 1: BBB+; Category 0: BBB-; The output value is the probability of being BBB+
Fu	1.398%	🔴	■ The fraction unbound in plasms<br>■ Low: <5%; Middle: 5~20%; High: > 20%

5. Metabolism

Property	Value	Comment
CYP1A2 inhibitor	0.809	■ Category 1: Inhibitor; Category 0: Non-inhibitor;<br>■ The output value is the probability of being inhibitor.
CYP1A2 substrate	0.951	■ Category 1: Substrate; Category 0: Non-substrate;<br>■ The output value is the probability of being substrate.
CYP2C19 inhibitor	0.927	■ Category 1: Inhibitor; Category 0: Non-inhibitor;<br>■ The output value is the probability of being inhibitor.
CYP2C19 substrate	0.829	■ Category 1: Substrate; Category 0: Non-substrate;<br>■ The output value is the probability of being substrate.
CYP2C9 inhibitor	0.79	■ Category 1: Inhibitor; Category 0: Non-inhibitor;<br>■ The output value is the probability of being inhibitor.
CYP2C9 substrate	0.934	■ Category 1: Substrate; Category 0: Non-substrate;<br>■ The output value is the probability of being substrate.
CYP2D6 inhibitor	0.481	■ Category 1: Inhibitor; Category 0: Non-inhibitor;<br>■ The output value is the probability of being inhibitor.
CYP2D6 substrate	0.947	■ Category 1: Substrate; Category 0: Non-substrate;<br>■ The output value is the probability of being substrate.
CYP3A4 inhibitor	0.771	■ Category 1: Inhibitor; Category 0: Non-inhibitor;<br>■ The output value is the probability of being inhibitor.
CYP3A4 substrate	0.415	■ Category 1: Substrate; Category 0: Non-substrate;<br>■ The output value is the probability of being substrate.

6. Excretion

Property	Value	Decision	Comment
CL	3.249	🔴	■ Clearance<br>■ High: >15 mL/min/kg; moderate: 5-15 mL/min/kg; low: <5 mL/min/kg
$T_{1/2}$	0.207	-	■ Category 1: long half-life ; Category 0: short half-life;<br>■ long half-life: >3h; short half-life: <3h<br>■ The output value is the probability of having long half-life.

7. Toxicity

Property	Value	Decision	Comment
hERG Blockers	0.725	🔴	■ Category 1: active; Category 0: inactive;<br>■ The output value is the probability of being active.
H-HT	0.083	🟢	■ Human Hepatotoxicity<br>■ Category 1: H-HT positive(+); Category 0: H-HT negative(-);<br>■ The output value is the probability of being toxic.
DILI	0.188	🟢	■ Drug Induced Liver Injury.<br>■ Category 1: drugs with a high risk of DILI; Category 0: drugs with no risk of DILI. The output value is the probability of being toxic.
AMES Toxicity	0.022	🟢	■ Category 1: Ames positive(+); Category 0: Ames negative(-);<br>■ The output value is the probability of being toxic.
Rat Oral Acute Toxicity	0.02	🟢	■ Category 0: low-toxicity; Category 1: high-toxicity;<br>■ The output value is the probability of being highly toxic.
FDAMDD	0.864	🔴	■ Maximum Recommended Daily Dose<br>■ Category 1: FDAMDD (+); Category 0: FDAMDD (-)<br>■ The output value is the probability of being positive.
Skin Sensitization	0.946	🔴	■ Category 1: Sensitizer; Category 0: Non-sensitizer;<br>■ The output value is the probability of being sensitizer.
Carcinogencity	0.028	🟢	■ Category 1: carcinogens; Category 0: non-carcinogens;<br>■ The output value is the probability of being toxic.
Eye Corrosion	0.003	🟢	■ Category 1: corrosives ; Category 0: noncorrosives<br>■ The output value is the probability of being corrosives.
Eye Irritation	0.065	🟢	■ Category 1: irritants ; Category 0: nonirritants<br>■ The output value is the probability of being irritants.

ADMETlab 2.0

Respiratory Toxicity	0.017	●	■ Category 1: respiratory toxicants; Category 0: respiratory nontoxicants<br>■ The output value is the probability of being toxic.

8. Environmental toxicity

Property	Value	Comment
Bioconcentration Factors	3.189	■ Bioconcentration factors are used for considering secondary poisoning potential and assessing risks to human health via the food chain.<br>■ The unit is –log10[(mg/L)/(1000*MW)]
IGC_{50}	5.099	■ Tetrahymena pyriformis 50 percent growth inhibition concentration<br>■ The unit is –log10[(mg/L)/(1000*MW)]
$LC_{50}FM$	7.049	■ 96-hour fathead minnow 50 percent lethal concentration<br>■ The unit is –log10[(mg/L)/(1000*MW)]
$LC_{50}DM$	6.92	■ 48-hour daphnia magna 50 percent lethal concentration<br>■ The unit is –log10[(mg/L)/(1000*MW)]

9. Tox21 pathway

Property	Value	Decision	Comment
NR-AR	0.113	●	■ Androgen receptor<br>■ Category 1: actives ; Category 0: inactives;<br>■ The output value is the probability of being active.
NR-AR-LBD	0.034	●	■ Androgen receptor ligand-binding domain<br>■ Category 1: actives ; Category 0: inactives;<br>■ The output value is the probability of being active.
NR-AhR	0.811	●	■ Aryl hydrocarbon receptor<br>■ Category 1: actives ; Category 0: inactives;<br>■ The output value is the probability of being active.
NR-Aromatase	0.758	●	■ Category 1: actives ; Category 0: inactives;<br>■ The output value is the probability of being active.
NR-ER	0.104	●	■ Estrogen receptor<br>■ Category 1: actives ; Category 0: inactives;<br>■ The output value is the probability of being active.
NR-ER-LBD	0.057	●	■ Estrogen receptor ligand-binding domain<br>■ Category 1: actives ; Category 0: inactives;<br>■ The output value is the probability of being active.
NR-PPAR-gamma	0.08	●	■ Peroxisome proliferator-activated receptor gamma<br>■ Category 1: actives ; Category 0: inactives;<br>■ The output value is the probability of being active.
SR-ARE	0.751	●	■ Antioxidant response element<br>■ Category 1: actives ; Category 0: inactives;<br>■ The output value is the probability of being active.
SR-ATAD5	0.438	●	■ ATPase family AAA domain-containing protein 5<br>■ Category 1: actives ; Category 0: inactives;<br>■ The output value is the probability of being active.

ADMETlab 2.0

SR-HSE	0.214		■ Heat shock factor response element<br>■ Category 1: actives ; Category 0: inactives;<br>■ The output value is the probability of being active.
SR-MMP	0.699		■ Mitochondrial membrane potential<br>■ Category 1: actives ; Category 0: inactives;<br>■ The output value is the probability of being active.
SR-p53	0.653		■ Category 1: actives ; Category 0: inactives;<br>■ The output value is the probability of being active.

10. Toxicophore Rules

**Property**	**Value**	**Comment**
Acute Toxicity Rule	0 alerts	■ 20 substructures<br>■ acute toxicity during oral administration
Genotoxic Carcinogenicity Rule	0 alerts	■ 117 substructures<br>■ carcinogenicity or mutagenicity
NonGenotoxic Carcinogenicity Rule	0 alerts	■ 23 substructures<br>■ carcinogenicity through nongenotoxic mechanisms
Skin Sensitization Rule	1 alerts	■ 155 substructures<br>■ skin irritation
Aquatic Toxicity Rule	0 alerts	■ 99 substructures<br>■ toxicity to liquid(water)
NonBiodegradable Rule	0 alerts	■ 19 substructures<br>■ non-biodegradable
SureChEMBL Rule	0 alerts	■ 164 substructures<br>■ MedChem unfriendly status

ADMETlab 2.0
